# Ataxia telangiectasia mutated (ATM) interacts with p400 ATPase for an efficient DNA damage response

**DOI:** 10.1186/s12867-016-0075-7

**Published:** 2016-11-04

**Authors:** Rebecca J. Smith, Matthew S. Savoian, Lauren E. Weber, Jeong Hyeon Park

**Affiliations:** Institute of Fundamental Sciences, Massey University, Palmerston North, New Zealand

**Keywords:** Phosphatidylinositol 3-kinase, Chromatin remodelling, DNA damage response, DNA repair, p400, ATM

## Abstract

**Background:**

Ataxia telangiectasia mutated (ATM) and TRRAP proteins belong to the phosphatidylinositol 3-kinase-related kinase family and are involved in DNA damage repair and chromatin remodeling. ATM is a checkpoint kinase that is recruited to sites of DNA double-strand breaks where it phosphorylates a diverse range of proteins that are part of the chromatin and DNA repair machinery. As an integral subunit of the TRRAP-TIP60 complexes, p400 ATPase is a chromatin remodeler that is also targeted to DNA double-strand break sites. While it is understood that DNA binding transcriptional activators recruit p400 ATPase into a regulatory region of the promoter, how p400 recognises and moves to DNA double-strand break sites is far less clear. Here we investigate a possibility whether ATM serves as a shuttle to deliver p400 to break sites.

**Results:**

Our data indicate that p400 co-immunoprecipitates with ATM independently of DNA damage state and that the N-terminal domain of p400 is vital for this interaction. Heterologous expression studies using Sf9 cells revealed that the ATM-p400 complex can be reconstituted without other mammalian bridging proteins. Overexpression of ATM-interacting p400 regions in U2OS cells induced dominant negative effects including the inhibition of both DNA damage repair and cell proliferation. Consistent with the dominant negative effect, the stable expression of an N-terminal p400 fragment showed a decrease in the association of p400 with ATM, but did not alter the association of p400 with TRRAP.

**Conclusion:**

Taken together, our findings suggest that a protein–protein interaction between ATM and p400 ATPase occurs independently of DNA damage and contributes to efficient DNA damage response and repair.

**Electronic supplementary material:**

The online version of this article (doi:10.1186/s12867-016-0075-7) contains supplementary material, which is available to authorized users.

## Background

Ataxia telangiectasia mutated (ATM) and TRRAP belong to the phosphoinositide 3-kinase-related protein kinase (PIKK) family and play a critical role in recognising a DNA double-strand break (DSB) site as well as in remodeling chromatin to facilitate DNA repair processes [1–4]. The kinase substrates of ATM include a wide variety of proteins that are critically required for DNA repair and check point control [5–9]. In addition to orchestrating the activity of DNA repair machineries, ATM can control chromatin structure indirectly through protein phosphorylation [10]. In its arguably most important function, activated ATM induces nuclear foci around the DSB site involving phosphorylation of H2A.X at serine 139 (γ-H2A.X) [3]. Histone γ-H2A.X has been used as an indicator of the degree of DNA damage as well as the effectiveness of the DNA repair process [11].

Localised chromatin relaxation around DSBs appears to be an ATM-independent process in which TRRAP participates through recruiting p400 and TIP60 to break sites [2, 12, 13]. TRRAP forms multi-subunit TRRAP-TIP60 complex (human NuA4 complex) containing the chromatin remodeler p400 and histone acetylase TIP60 as an integral subunit where TRRAP, p400 and TIP60 are all required for DNA damage-induced chromatin decondensation [14–16]. The mechanism of how p400 is targeted to the DSB is poorly understood but the interaction of p400-containing complexes with DNA repair proteins such as Mdc1 and Rad51 have been suggested as a recruitment mechanism [17]. Taken together, DSBs seem to induce chromatin relaxation in both ATM-dependent and independent manners. Importantly, ATM and TRRAP appear to act independently for efficient DNA repair by targeting different enzyme activities to the DSB sites. ATM and TRRAP have a modular domain structure, with a common FRAP-ATM-TRRAP-C-terminal (FATC) domain that is interchangeable in terms of the interaction ability with TIP60 [18]. The swappable domain of PIKK proteins suggests that there may be additional binding partners shared between members of the PIKK family. Here we demonstrate that the p400 ATPase, which has previously been shown to interact with TRRAP [19, 20] associates with ATM independently of DNA damage, providing an additional mechanism of ATM-dependent chromatin relaxation and p400 targeting.

## Results

### The p400 associates with ATM in HEK293T cells

**Fig. 1:**
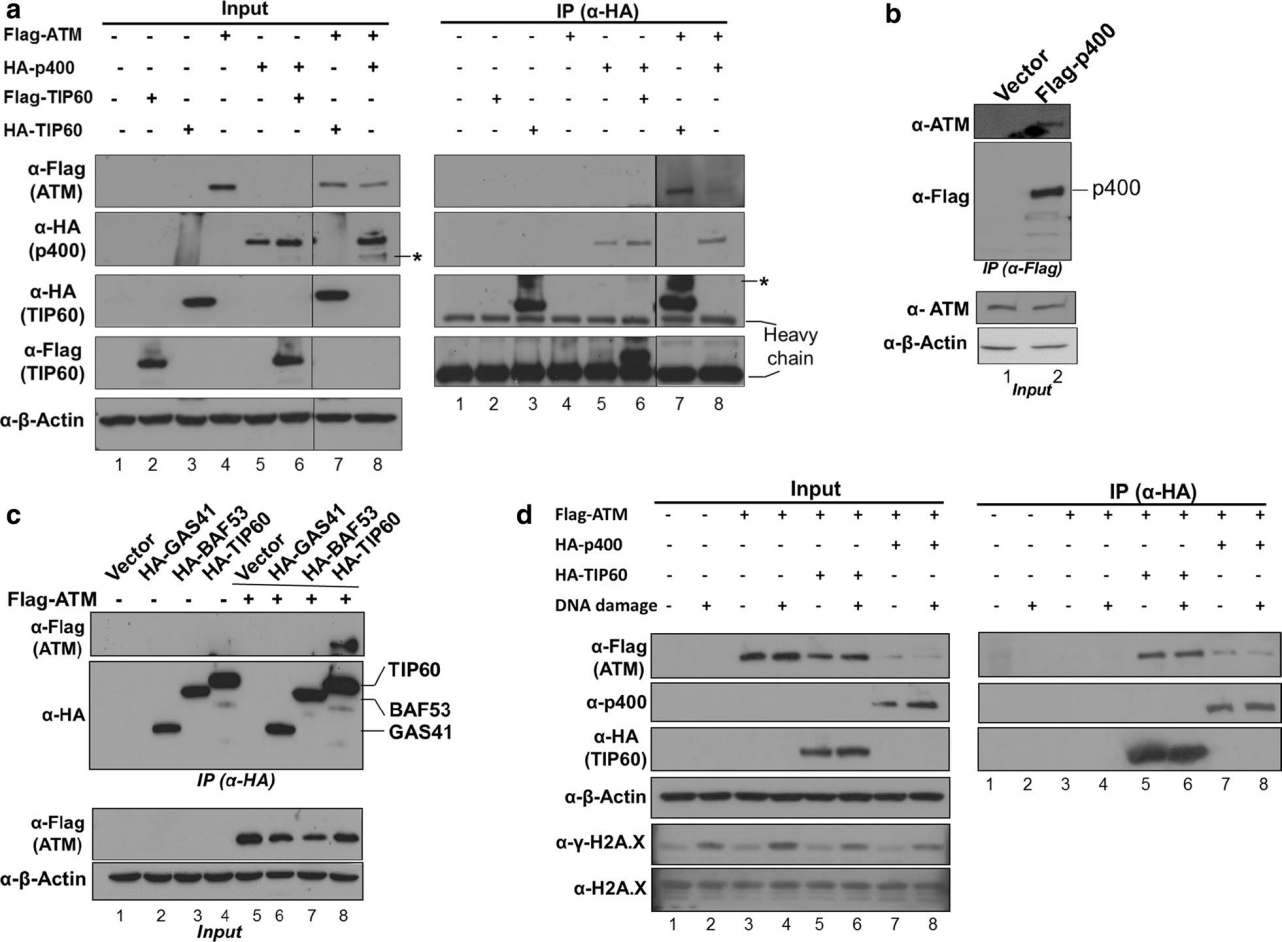
Co-immunoprecipitation assays for the interaction of ATM and p400. **a** HEK293T cells were transfected with pcDNA-Flag-ATM, CβS-HA-TIP60, CβF-TIP60 and CβS-HA-p400 in various combinations. Both input and anti-HA immunoprecipitation samples were analysed on an immunoblot using anti-HA and anti-Flag antibodies while the input samples were additionally examined with anti-actin antibody as a loading control. Immunoglobulin heavy chain is shown in the *right panel* of immunoprecipitation analysis. * non-specific band. **b** HEK293T cells were transfected with plasmid expressing Flag-p400 (*lane 2*). The whole cell extracts were subjected to immunoprecipitation with M2 agarose and the immunoblot was examined with anti-ATM and anti-Flag antibodies. The 5% (v/v) of input whole cell extract was also analysed by immunoblotting in the* bottom panels* to show the total ATM level and β-actin. **c** HEK293T cells were transfected with pcDNA-Flag-ATM, CβS-HA-TIP60, CβS-HA-GAS41, CβS-HA-BAF53 in various combinations. Both input and immunoprecipitation samples were examined by immunoblotting. **d** Co-immunoprecipitation between ATM and p400 in the presence and absence of DNA damage. HEK293T cells were transfected in duplicate with pcDNA-Flag-ATM alone or together with CβS-HA-TIP60 or CβS-HA-p400. Transfected cells were incubated in the presence and absence of bleomycin for 4 h before being analysed by immunoblotting. To assess the DNA damage by bleomycin, histones were separately prepared by acid extraction and the immunoblot was analysed by anti-γ-H2A.X (phospho-S139) antibody

The conserved structural features of ATM and TRRAP suggest that they interact with various proteins in common for their specific functions in DNA damage response and transcriptional regulation [1]. To examine whether p400 associates with ATM, similar to its interaction with TRRAP, co-immunoprecipitation assays were performed using different epitope tagged ATM, p400 and TIP60 proteins (Fig. 1a). The association of TIP60 with either ATM or p400 has previously been described and was used here as positive controls (Fig. 1a, right panel, lanes 6 and 7) [19, 21]. Despite its low expression relative to HA-TIP60, HA-p400 was also able to co-immunoprecipitate with Flag-ATM (Fig. 1c, right panel, lanes 7 and 8). This raises the possibility that p400 could be a novel interacting partner of ATM in addition to TIP60. The interaction studies using endogenous ATM and p400 have not been successful mainly due to the lack of immunoprecipitation grade antibodies and relatively low expression levels of these high molecular weight proteins. However, Flag-p400 ectopically expressed in HEK293T cells was able to precipitate endogenous ATM (Fig. 1b). As the interaction of p400 with ATM occurs in the endogenous level of ATM, the interaction would be physiologically relevant.

To examine the possibility that the association of p400 with ATM occurs indirectly through the multi-subunit TRRAP-TIP60 complex, co-immunoprecipitation experiments were performed between ATM and two integral subunits of the TRRAP-TIP60 complex, BAF53 and GAS41 (Fig. 1c) [22, 23]. HEK293T cells were transfected with plasmids expressing Flag-ATM, HA-TIP60, HA-GAS41 and HA-BAF53 in various combinations and the whole cell extract was subjected to immunoprecipitation using anti-HA antibody-conjugated beads. The input and immunoprecipitation samples were analysed by immunoblotting using anti-HA and anti-Flag antibodies. The result showed that HA-TIP60 immunoprecipitated with Flag-ATM as expected whereas the other two members of the TRRAP-TIP60 complex did not associate, even under a forced overexpression setting (Fig. 1c, lane 8 versus lanes 6, 7). This result would suggest that the interaction of p400 with ATM might occur independently from the previously identified nuclear complexes containing GAS41 or BAF53.

Both ATM and p400 are targeted to the site of DSBs and play a critical role in DNA repair [3, 12, 15, 24]. To investigate whether the interaction between ATM and p400 is dependent on DNA damage, HEK293T cells were transfected in duplicate with Flag-ATM alone or together with either HA-TIP60 or HA-p400 and the interaction was examined in the presence and absence of bleomycin, an inducer of DSBs (Fig. 1d). To obtain comparable co-expression levels of Flag-ATM and HA-p400 in HEK293T cells, higher molar ratio of plasmid DNA expressing p400 was used for the transient co-expression and thereby resulted in a less expression of Flag-ATM in cells (Fig. 1d, lanes 7, 8). As expected, bleomycin induced an increase in H2A.X phosphorylation but did not alter the expression levels of ATM, p400 or TIP60 within the duplicate of transfection (Fig. 1d, left panel). Consistent with a previous report, the levels of ATM associated with TIP60 were remained similar regardless of DNA damage (Fig. 1d, right panel, compare lanes 5, 6) [25]. Similarly, the level of ATM associated with p400 did not change significantly in the presence of DNA damage (Fig. 1d, right panel, compare lanes 7, 8), suggesting that a minor portion of ATM is associated with p400 irrespective of DNA damage.

### The interaction between p400 and ATM can be reconstituted in insect cells

**Fig. 2:**
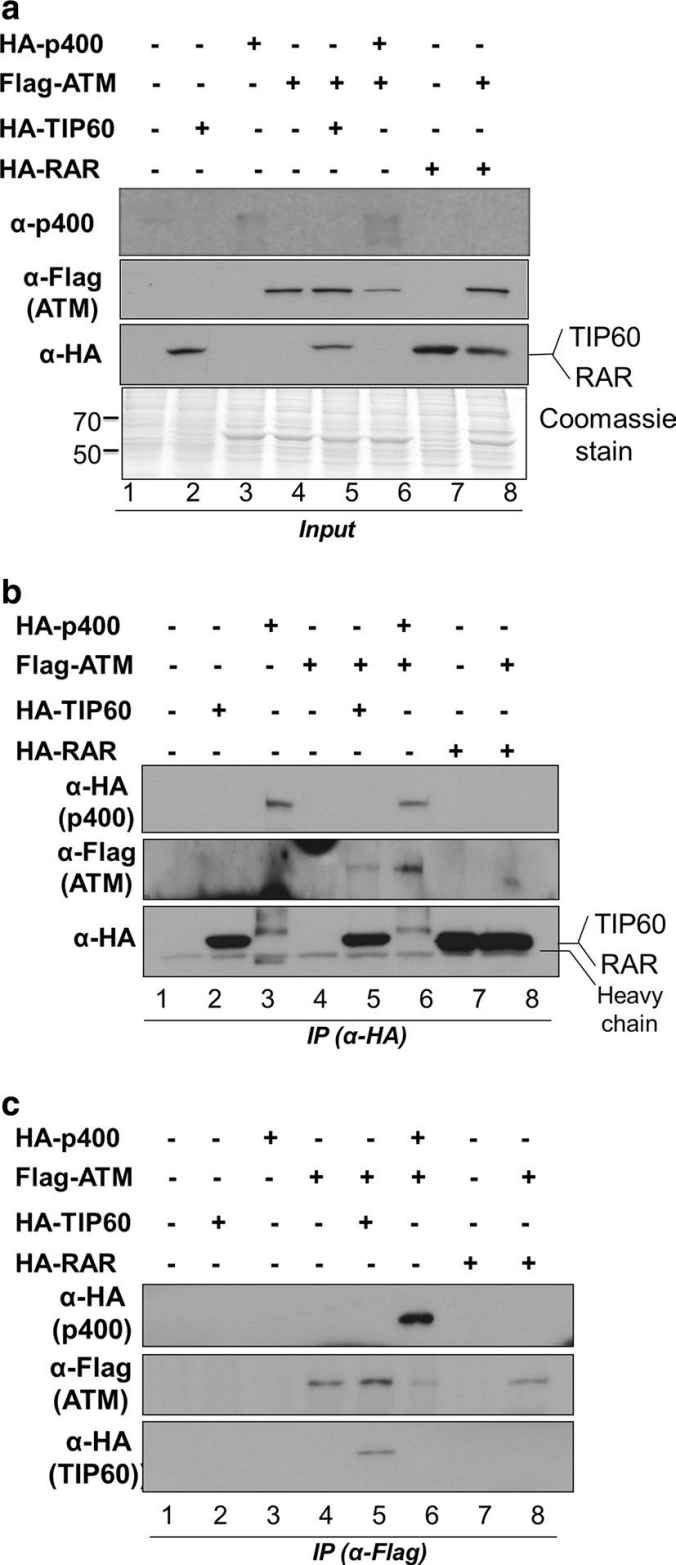
Heterologous expression of ATM and p400 in Sf9 cells and their reciprocal interaction assays. **a** Cell lysate were prepared at 48 h post-infection and analysed by immunoblot or by Coomassie Brilliant Blue R-250 staining. **b**, **c** Equal volume of cell lysate from A was subjected to immunoprecipitation with anti-HA antibody conjugated beads (**b**) and anti-Flag antibody conjugated M2 agarose (**c**), respectively

The common recruitment of ATM and multiple p400-containing complexes to the site of DNA damage makes it technically challenging to characterise p400-containing nuclear complexes at the DSB sites. To overcome such ambiguous localisation of multi-subunit p400 complexes and exclude the possibility of bridging proteins for the ATM interaction, we employed a heterologous gene expression system in baculovirus-infected Sf9 cells (Fig. 2). HA-RAR is the HA-tagged nuclear retinoic acid receptor and was used as a negative control to confirm that the interaction observed between Flag-ATM and HA-p400 was not dependent on the HA tag or an overexpression-derived artefact. The cells were harvested at 48 h post-infection to prepare whole cell extracts. Expression of recombinant proteins was confirmed by immunoblotting (Fig. 2a). HA-p400 was expressed at a low level with significant protein degradation or possibly truncated protein expression which was not improved by altering the time course of post infection. It is likely due to the large size and instability of the recombinant protein (Fig. 2a, lanes 3, 6). Therefore, higher viral titre of baculovirus expressing p400 was used to compensate a low expression level in which the co-expression level of Flag-ATM was reduced proportionally (Fig. 2a, lane 6). The whole cell extracts were divided into two equal volumes and subjected to immunoprecipitation using anti-HA antibody-conjugated beads (Fig. 2b) or anti-Flag antibody-conjugated M2 agarose (Fig. 2c). Figure 2b shows that HA-p400 and HA-TIP60 co-immunoprecipitated with Flag-ATM respectively, while significantly overexpressed HA-RAR did not. Given that HA-p400 is expressed at markedly lower levels than HA-TIP60, the association of p400 with ATM appears more efficient than that of TIP60 in Sf9 cells (Fig. 2b, compare Flag-ATM levels in lanes 5, 6). The interaction was further confirmed by performing the reciprocal immunoprecipitation using M2 agarose (Fig. 2c). The result reveals that HA-p400 co-purifies with ATM, similar to the result observed with HA-TIP60 (Fig. 2c, lanes 5, 6). Given that ATM and p400 were exclusively cytoplasmic in Sf9 cells for this interaction (Additional file 1: Figure S1), the possibility that both proteins bind to DNA and co-immunoprecipitate through indirect DNA bridges was excluded. Taken together, the reciprocal co-immunoprecipitation of ATM and p400 in Sf9 cells would suggest a protein–protein interaction which is comparable to or more efficient than the previously identified association of ATM and TIP60 [15].

### The N-terminal fragments of p400 interact with ATM

**Fig. 3:**
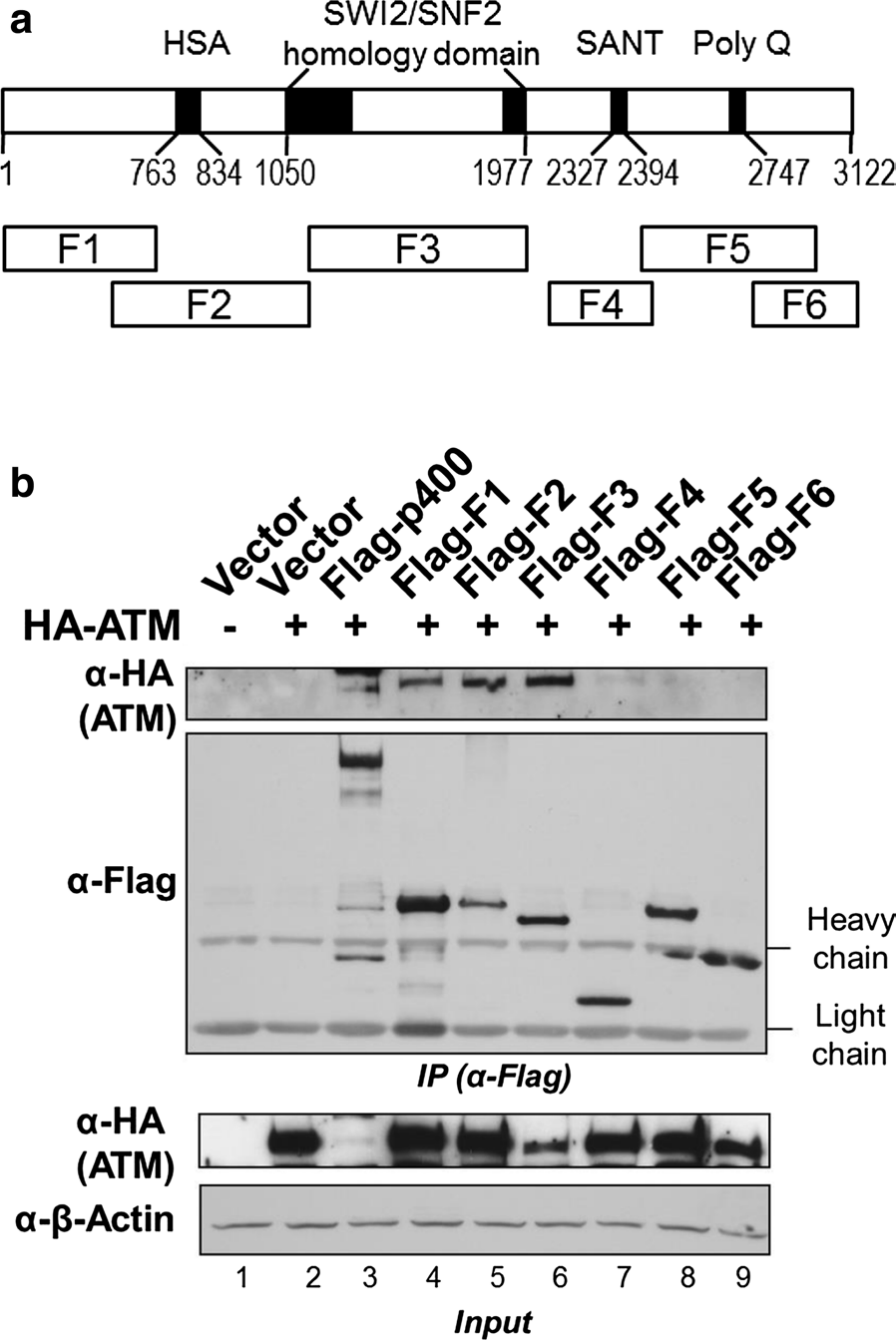
ATM interaction domain mapping of p400. **a** Schematic presentation of the conserved domains on the p400 protein. HSA, Helicase/SANT-associated domain; SANT, SWI3-ADA2-N-CoR-TFIIIB domain. **b** Co-immunoprecipitation of ATM with fragment 1 (*F1*) to fragment 6 (*F6*) of p400. HEK293T cells were transfected with plasmids expressing HA-ATM alone or together with p400 and its derivatives

To map the ATM interaction region on the p400 polypeptide, co-immunoprecipitation assays were performed using various p400 fragments expressed in HEK293T cells. A schematic representation of the p400 derivatives used in this mapping is shown in Fig. 3a. HA-ATM and Flag-p400 derivatives were transiently expressed in HEK293T cells and the whole cell extracts were subjected to immunoprecipitation by M2 agarose (Fig. 3b). Ectopic HA-ATM expression levels varied among immunoprecipitated samples. Despite the effort to get similar levels of protein expression, ectopic ATM expression was barely detectable when co-expressed with full length Flag-p400 protein (Fig. 3b, input ATM panel, lane 3). To map the ATM interaction within p400, six sequential Flag-tagged fragments that spanned the p400 protein were used in co-immunoprecipitation experiments (Fig. 3b, IP panel, lanes 3–9). As expected, full length p400 co-immunoprecipitated with ATM but at a low level, which would result from the poor expression of ATM seen in the input sample (Fig. 3b, lane 3). The large band seen in lane 3 of both anti-HA panels is derived from residual staining of Flag-p400 after insufficient stripping of the membrane prior to incubation with the anti-HA antibody. The three fragments spanning the N-terminal half of p400 were each able to associate with ATM (Fig. 3b, lanes 4–6). The association of multiple domains of p400 with ATM suggests that the interaction involves a direct protein interaction as well as indirect associations through bridging proteins. The F4 fragment of p400 was not associated with ATM but has been shown to interact with TIP60 previously, suggesting that the ATM-p400 interaction occurs in a manner distinct to that observed in the TIP60-p400 interaction [19].

### The expression of ATM-interacting fragments of p400 increases susceptibility to bleomycin without affecting ATM phosphorylation

**Fig. 4:**
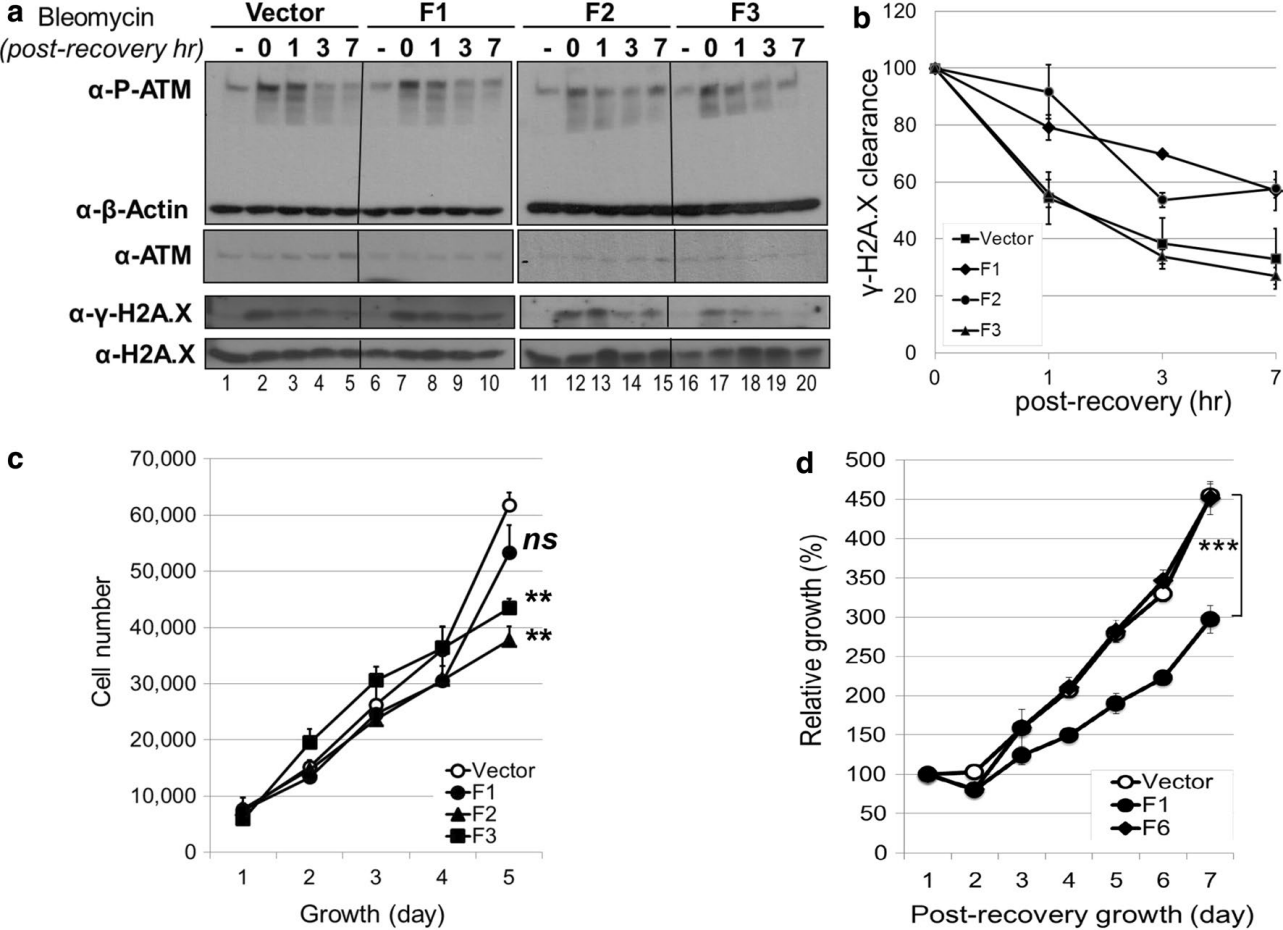
Bleomycin susceptibility assays for the ectopic expression of ATM-interacting p400 fragments. **a** Time course of DNA damage response after bleomycin exposure. Puromycin-selected U2OS cells were exposed to bleomycin at 10 μg/ml for 1 h, replaced with normal growth medium and incubated further for an indicated period as a post-recovery time. Whole cell extracts and histone extracts were analysed by immunoblotting with antibodies as indicated. **b** γ-H2A.X clearance after bleomycin exposure. The signals of γ-H2A.X to total H2A.X on the immunoblot in (**a**) were quantified in triplicate with Image J software. The peaked ratio right after the bleomycin treatment (0 h post-recovery) set to 100 and the relative changes were calculated. **c** Growth curve of puromycin-selected U2OS cells. U2OS cells were infected with lentivirus expressing Flag-F1, Flag-F2 or Flag-F3, and selected for 5 days using puromycin. Each cell line was seeded in 96-well plate in triplicate and monitored for the cell proliferation. (Growth day 5: n = 3, *p < 0.05, **p < 0.01, ns: not significant by the Student *t* test compared to the vector). The experiments were repeated triple times with similar results. **d** Bleomycin sensitivity assay of U2OS cells. U2OS cells stably expressing Flag-F1 or Flag-F6, were exposed to bleomycin at 10 μg/ml for 12 h and seeded in 96-well plate in triplicate to assess the bleomycin sensitivity. The cell number observed at the first day of post-recovery set to 100% and the relative cell growth was calculated during the post-recovery period. (Post-recovery day 7: n = 3, ***p < 0.001)

The depletion of p400 protein or forced overexpression of a catalytically-null p400 revealed that p400 is required for decondensation of chromatin and subsequent recruitment of repair proteins [12, 17]. However, functional analysis of the ATM-p400 complex using siRNA mediated knockdown has been complicated as it does not differentiate a defective human NuA4 complex from a unique ATM-p400 association. The specific interaction between ATM and the N-terminal fragments of p400 suggests that it might provide a novel tool to study the DNA damage response. To this end, lentivirus was used to stably express the different p400 fragments in U2OS cells (Fig. 4, Additional file 2: Figure S2). To examine whether the expression of p400 fragments alters ATM activation and H2A.X phosphorylation after DNA damage, U2OS cells were exposed to the DNA damaging agent, bleomycin. The whole cell extracts were prepared at different post-recovery times after bleomycin treatment and the phosphorylation status of ATM and H2A.X was examined by immunoblotting (Fig. 4a). U2OS cells infected by control lentivirus (vector in Fig. 4a) showed a peak of ATM phosphorylation immediately after bleomycin treatment (Fig. 4a, lane 2) and returned to the basal level within a 3 h recovery period (Fig. 4a, P-ATM panel, compare lane 3 versus lane 1). Similar observations were made in each of the three U2OS cell lines expressing F1, F2 or F3 fragments. This result indicates that the ectopic expression of p400 fragments does not affect ATM autophosphorylation or the activation process. The clearance of ATM-mediated H2A.X phosphorylation is an indicator of how efficiently DNA damage is repaired. U2OS cells infected by control lentivirus (vector) showed a rapid clearance of γ-H2A.X during a recovery period in which γ-H2A.X returned to a near basal level within 7 h (Fig. 4a, γ-H2A.X panel, compare lane 5 versus lane 1). U2OS cells expressing F3 displayed a similar pattern to the vector control whereas cells expressing either of F1 or F2 fragments showed a slower clearance of γ-H2A.X (Fig. 4b). These results indicate that the ectopic expression of ATM-interacting p400 fragments interferes with the clearance of γ-H2A.X and hinders DNA damage repair.

To determine if the ectopic expression of ATM-interacting p400 fragments has any cytotoxic effect on normal cellular growth, cell numbers were monitored for 5 days in normal growth condition (Fig. 4c). U2OS cells expressing the F2 or F3 fragment showed a retarded growth compared to the control virus-infected cells. Given that the long-term stable expression levels of F2 and F3 fragments are much lower than that of transient transfection (Additional file 2: Figure S2A), cells with lower expression levels of these fragments appear to be preferentially selected for during the course of experiments. To examine whether the ectopic expression of an ATM-interacting p400 fragment affects drug susceptibility, the F1 and F6 fragments that did not inhibit cellular proliferation at a comparable expression level were tested for bleomycin susceptibility assay (Fig. 4d and Additional file 2: Figure S2B). U2OS cells were exposed to bleomycin for 12 h and the cellular growth was monitored for 7 days. While the cells infected with virus for vector control or F6 fragment showed a comparable recovery and growth, the ectopic expression of ATM-interacting F1 fragment resulted in severely restricted growth during the post-recovery period.

**Fig. 5:**
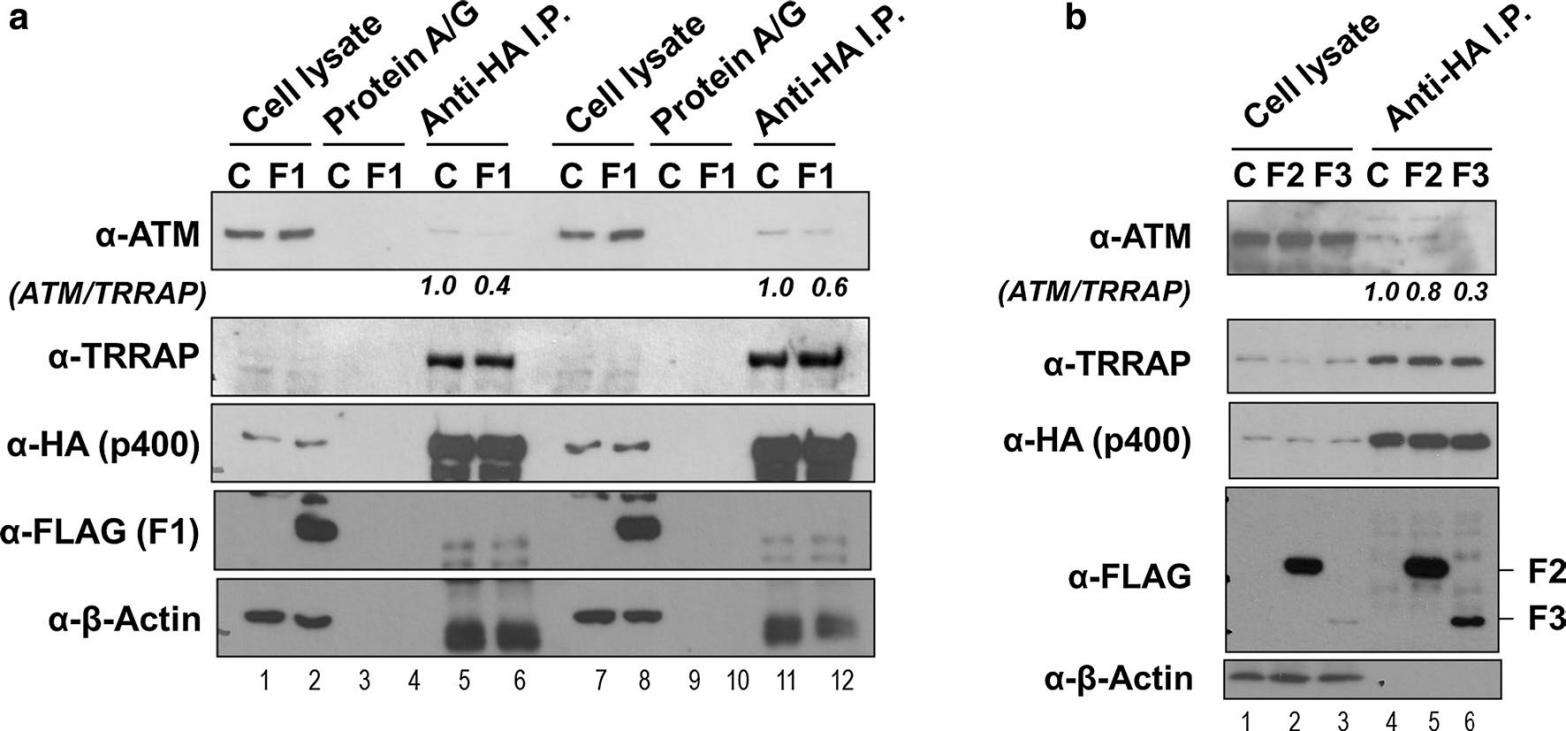
The N-terminal fragments of p400 interfere with the protein–protein interaction between ATM and p400. **a** HEK293T cells virally transduced by either control or F1 fragment expressing lentivirus were selected against puromycin and then used for the transient expression of HA-p400 in duplicate. The cell lysates were pre-cleared by protein A/G beads for 3 h and subjected to immunoprecipitation by anti-HA antibody-conjugated beads. Two percent of prepared cell lysates was used as input to ensure comparable expression levels of ATM, TRRAP and p400 in control (C) and Flag-F1 fragment (F1) cell lines. The immunoblot signals from co-immunoprecipitated ATM and TRRAP were quantified using Image J software and the ratios of ATM to TRRAP were calculated (ATM/TRRAP). **b** The same experiments were conducted except the transient expression of F2 and F3 fragments in HEK293T cells. The *data* represents one of two independent experiments with similar results

To investigate how the ectopic expression of F1 fragment distinctively affects the ATM-p400 interaction from the TRRAP-p400 complex, HEK293T cell line stably expressing Flag-F1 was generated and used to compare the amount of co-immunoprecipitated ATM and TRRAP with p400, respectively. Transient expression of HA-p400 was performed in duplicate in HEK293T cell lines (Fig. 5a, lanes 1–12). The cell lysates were pre-cleared by protein A/G beads and subjected to immunoprecipitation using anti-HA antibody-conjugated beads. As expected, the amount of TRRAP was significantly enriched by HA-p400 immunoprecipitation but was not altered by F1 fragment expression (Fig. 5a, lanes 5–6 and 11–12 of the α-TRRAP panel). However, the amount of p400-associated ATM was consistently reduced in the presence of F1 expression (Fig. 5a, lanes 5–6 and 11–12 of the α-ATM panel), suggesting that F1 interferes with the protein–protein interaction between ATM and p400.

In addition to the strong association with ATM in our studies, the F2 and F3 fragments covering helicase/SANT-associated (HSA) and SWI/SNF2 homology domains have previously been shown to interact with various subunits of NuA4 complex and serve as a critical core for the complex assembly [20, 26]. Therefore, similar interference studies were done to investigate whether the overexpressed F2 or F3 affects the ATM-p400 interaction (Fig. 5b). The result shows that F3 fragment, albeit its minimal expression level, significantly reduced the amount of ATM associated with p400, suggesting that the SWI/SNF2 homology domain of p400 would also specifically interfere with the protein–protein interactions of ATM-p400 (Fig. 5b, lane 6 of anti-ATM immunoblot). In contrast, neither of the fragments affects the amount of co-immunoprecipitated TRRAP by HA-p400 (Fig. 5b, lanes 4–6 of anti-TRRAP immunoblot). Interestingly, both F2 and F3 but not F1 were co-immunoprecipitated with HA-p400, implying that various NuA4 subunits that interact with F2 or F3 might play a role as a bridging protein to bring a full-length p400 together with its fragments (Fig. 5b, lanes 5–6 of anti-Flag immunoblot).

Taken together, ectopic expression of the N-terminal fragments of p400 lead to more severe drug-induced cytotoxicity or retarded cellular growth, and the underlying mechanism might involve the interference of p400 function with ATM or human NuA4 complexes.

## Discussion

Here we report that a catalytic subunit of the TRRAP-TIP60 complex, p400 can associate with ATM in both HEK293T and Sf9 cells. The association between ATM and p400 is not surprising since FATC domains of ATM and TRRAP are functionally exchangeable for interaction partners such as TIP60 [18]. The MRN (Mre11-Rad50-Nbs1) complex has also been shown to interact physically and functionally with both ATM and TRRAP during DSB end joining processes [27]. The protein–protein interaction between ATM and p400 appears to occur independently of DNA damage and the interacting p400 complex would be unique since the integral subunits of TRRAP-TIP60 complexes, BAF53 and GAS41 did not associate with ATM. Despite the reciprocal interaction of ATM and p400 in Sf9 cells, we cannot exclude a possibility that the association of p400 with ATM is mediated by indirect associations through unknown insect bridging proteins. It is important to note that in this study the interaction between ATM and p400 was seen mainly through the use of overexpressed proteins, although Flag-tagged p400 was able to precipitate endogenous ATM (Fig. 1b). The failure to co-immunoprecipitate the two proteins endogenously is consistent with a recent analysis of endogenous p400 and ATM complexes that did not show the stoichiometric association of p400 or TIP60 [28]. Therefore, it is possible that the interaction between ATM and p400 seen in this study may represent a transient interaction only present in a certain cellular condition or derived from a minor fraction of the endogenous complexes.

Suppression of the ATM-p400 interaction appears to inhibit DSB repair as shown through our experiments using stably expressed p400 fragments in U2OS cells. However, clear functional roles in the DNA repair process remain to be elucidated. We speculate that the ATM-p400 association could influence the DNA damage response in two different ways either regulating ATM activity or targeting the p400 protein to the DSB site. Firstly, the p400 protein could regulate the activity of ATM or ATM-associated TIP60. In support of this, p400 is known to be a negative regulator of TIP60 when they are within the same TRRAP-TIP60 complex [19]. Previously, ATM activation has been shown to be more robust and rapid in p400-depleted cells, suggesting that p400 is a negative regulator of ATM activation [17]. More importantly, we show that the interaction between ATM and p400 is not altered regardless of DNA damage, suggesting that the inhibitory effects on ATM would be regulated by post-translational modifications [29]. As a second functional role, ATM may serve to ferry p400 to locations of DNA damage. This speculation would explain a previous observation that the loss of ATM reduces or delays histone loss at the DSB site [30]. The association of ATM-p400 may allow a rapid recruitment of p400 activity to the DNA double-strand break site as ATM is one of the earliest DNA repair proteins found at the DNA damage sites [31]. By increasing the local concentration of p400 together with TRRAP and TIP60 at the DNA break site, dynamic complex assembly would be achieved for efficient chromatin remodeling activity [12].

Our results show that N-terminal fragments of p400 interact with ATM in a similar but distinct manner to that observed in the p400-TIP60 interaction. In particular, stable expression of p400 fragments containing the helicase-SANT–associated (HSA) domain (F2) or the SWI/SNF homology domain (F3) in U2OS cells are inhibitory to the cellular growth. The F2 and F3 regions of p400 have been shown to be important in the integrity and assembly of the TRRAP-TIP60 complex [20, 26, 32]. Given that the functional loss of TRRAP induces cell proliferation arrest [33], it is intriguing to know whether the F2 or F3 region of p400 would directly interfere with the function of TRRAP-TIP60 complexes and cause a growth inhibitory effect. On the other hand, the stable expression of the N-terminal F1 fragment (1–719) of p400 did not interfere with normal cellular growth but sensitised the cells upon bleomycin treatment. F1 does not have any conserved domain that has been previously shown to interact with other chromatin remodeling complexes or DNA repair proteins. Therefore, the F1 fragment-induced sensitisation in U2OS cells is unlikely to be due to the inhibition of the TRRAP-TIP60 complex or changes in gene expression, but more likely caused by interfering with the interaction between ATM and p400 as shown in Fig. 5a. A dominant negative approach using the F1 fragment will be useful to further dissect the complicated functions carried out by multiple p400 complexes.

## Conclusion

ATM interacts with p400 independently of DNA damage in HEK293T cells and their interaction may represent a transient minor interaction under a certain physiological condition. The N-terminal domains of p400 is vital for the interaction with ATM. Based on these findings, we have shown that the expression of the N-terminal p400 fragment sensitizes U2OS cells to bleomycin.

## Methods

### Cell culture

HEK293T and U2OS cells were originally obtained from ATCC (Rockville, MD). Cells were routinely maintained on a regular basis in the presence of mycoplasma preventive antibiotics, plasmocin (5 µg/ml, Invivogen) and confirmed for the absence of mycoplasma contamination. HEK293T and U2OS cells were maintained in Dulbecco’s modified Eagle’s medium (Gibco/Life Technology) containing 10% fetal calf serum (FCS, Sigma Aldrich) and supplemented with 50 U/ml penicillin, 50 μg/ml streptomycin and 0.125 µg/ml amphotericin B (Sigma Aldrich) and cultured at 37  °C in a 5% CO_2_ humidified incubator. Sf9 cells were cultured in normal atmospheric conditions in supplemented Grace’s Insect Media (Gibco/Life Technology) with 10% FCS (Sigma), 0.1% Pluronic acid (Sigma), 10 µg/ml gentamycin, 50 U/ml penicillin, 50 μg/ml streptomycin and 0.125 µg/ml amphotericin B (Sigma Aldrich). Baculovirus and lentivirus were prepared using methods described in the manufacturer’s procedure (Invitrogen and System Biosciences, respectively). For the analysis of cellular proliferation, the Cyquant cell proliferation assay kit (Invitrogen, C7026) was used as described previously [34].

### Plasmid and antibodies

The cDNA of Flag-tagged ATM was obtained from Dr Chris Bakkenist (University of Pittsburg, USA). Mammalian expression vectors for p400, p400-derived fragments (F1–F6), TIP60, BAF53 and GAS41 were described in previous studies [19, 23, 35, 36]. All other plasmids in this study including baculoviral and lentiviral expression vectors were constructed by standard cloning procedures. The primary antibodies used in this study were H2AX (Milipore, 07-627), H2AX S139P (Milipore, 07-164), β-actin (Sigma, A5316), ATM (Calbiochem, 819844), P-ATM (Serine 1981, Abcam, ab36810), Flag M2 (Sigma, F3165), and HA (Sigma, SAB4300603). Antibodies against TIP60 and p400 were produced from rabbits and have been used in previous studies.

### Protein extraction, immunoprecipitation and immunoblotting

HEK293T and Sf9 cells were harvested by scraping into PBS, pelleted and resuspended in lysis buffer (20 mM HEPES pH 7.4, 120 mM NaCl, 1.5 mM MgCl_2_, 1 mM EGTA, 50 mM NaF, 0.2% Tween-20, 0.5 mM DTT, 1 mM PMSF). The lysed supernatant was used as whole cell extracts. To extract histones from the insoluble fraction, the pellet after soluble protein extraction was resuspended in 1 ml H_2_O then centrifuged at 16,000*g* for 10 min. The supernatant was aspirated and the pellet was resuspended in 200 µl of 0.2 N HCl and left on ice overnight. The sample was centrifuged at 16,000*g* for 10 min and the supernatant was transferred to a new microfuge tube before being neutralised with 10 µl of 2 M Tris, pH 10. Anti-Flag (M2) affinity gel (Sigma) or EZview™ Red Anti-HA affinity gel (Sigma) were added to the cell lysate and incubated overnight at 4 °C. Beads were washed and analysed by an SDS-PAGE on a 5–10% two step gradient gel followed by the immunoblot procedure.

## Additional files

**Additional file 1: Figure S1.** Sf9 cells were infected with the baculovirus expressing indicated heterologous transgenes and cultured for 24, 36 or 48 h before processing. (**A**) Flag-ATM (red) is found in the nucleus (identified by DAPI staining; blue) as well as in the cytosplasm (cell boundaries are visible in the DIC image) at all-time points. (**B**) HA-p400 (green) is also distributed in all cellular compartments 24 h after infection. As time advances (36, 48 h), the protein exits from the nucleus (delineated by DAPI; blue) until it is exclusively cytoplasmic. (**C**) cells co-expressing Flag-ATM (red) and HA-p400 (green) exhibit dynamic protein relocalisation. 24 h post-infection a common pool of ATM and p400 is observed in the nucleus (DAPI; blue). The nuclear localisation of both proteins decreases with a concomitant enrichment of the cytoplasmic fraction (36, 48 h). All scale bars are 5 µm.

**Additional file 2: Figure S2.** (**A**) ectopic expression of ATM-interacting p400 fragments in U2OS cells. U2OS cells were infected with lentivirus expressing Flag-tagged p400 fragments and selected for 5 days against puromycin. Total 500 μg of protein was immunoprecipitated with M2 agarose and analysed by immunoblotting with anti-Flag antibody. (**B**) growth curve of puromycin-selected U2OS cells. U2OS cells were infected with lentivirus expressing Flag-F1or Flag-F6, and selected for 5 days against puromycin. Each cell line was seeded in 96-well plate in triplicate and monitored for the cell proliferation.

## Abbreviations

ATM: ataxia telangiectasia mutated
TRRAP: transformation/transcription domain associated protein
PIKK: phosphoinositide 3-Kinase-related protein kinase
DSB: DNA double-strand break
NuA4: nucleosome acetyltransferase of H4
FATC: FRAP-ATM-TRRAP-C-terminal
HEK293T: human embryonic kidney 293T
RAR: retinoic acid receptor
HSA: Helicase/SANT-Associated
SANT: SWI/SNF2, Ada, N-CoR, TFIIIB
SWI/SNF2: SWItch, sucrose non-fermentable
MRN: Mre11-Rad50-Nbs1
FCS: fetal calf serum
PBS: phosphate-buffered saline
HEPES: 4-(2-hydroxyethyl)-1-piperazineethanesulfonic acid
EGTA: ethylene glycol tetraacetic acid
DTT: Dithiothreitol
PMSF: phenylmethane sulfonyl fluoride
SDS-PAGE: sodium dodecyl sulfate polyacrylamide gel electrophoresis
DAPI: 4′,6-Diamidino-2-Phenylindole
