# Supplementary material for: Ataxia telangiectasia mutated (ATM) interacts with p400 ATPase for an efficient DNA damage response
Source: BMC Mol Biol. 2016 Nov 4;17:22. doi: 10.1186/s12867-016-0075-7 (PMC5097431; doi:10.1186/s12867-016-0075-7)
Supplement: Supplementary file 1 — Additional file 1: Figure S1. Sf9 cells were infected with the baculovirus expressing indicated heterologous transgenes and cultured for 24, 36 or 48 h before processing. (A) Flag-ATM (red) is found in the nucleus (identified by DAPI staining; blue) as well as in the cytosplasm (cell boundaries are visible in the DIC image) at all-time points. (B) HA-p400 (green) is also distributed in all cellular compartments 24 h after infection. As time advances (36, 48 h), the protein exits from the nucleus (delineated by DAPI; blue) until it is exclusively cytoplasmic. (C) cells co-expressing Flag-ATM (red) and HA-p400 (green) exhibit dynamic protein relocalisation. 24 h post-infection a common pool of ATM and p400 is observed in the nucleus (DAPI; blue). The nuclear localisation of both proteins decreases with a concomitant enrichment of the cytoplasmic fraction (36, 48 h). All scale bars are 5 µm. [file 12867_2016_75_MOESM1_ESM.pptx]

## Slide 1
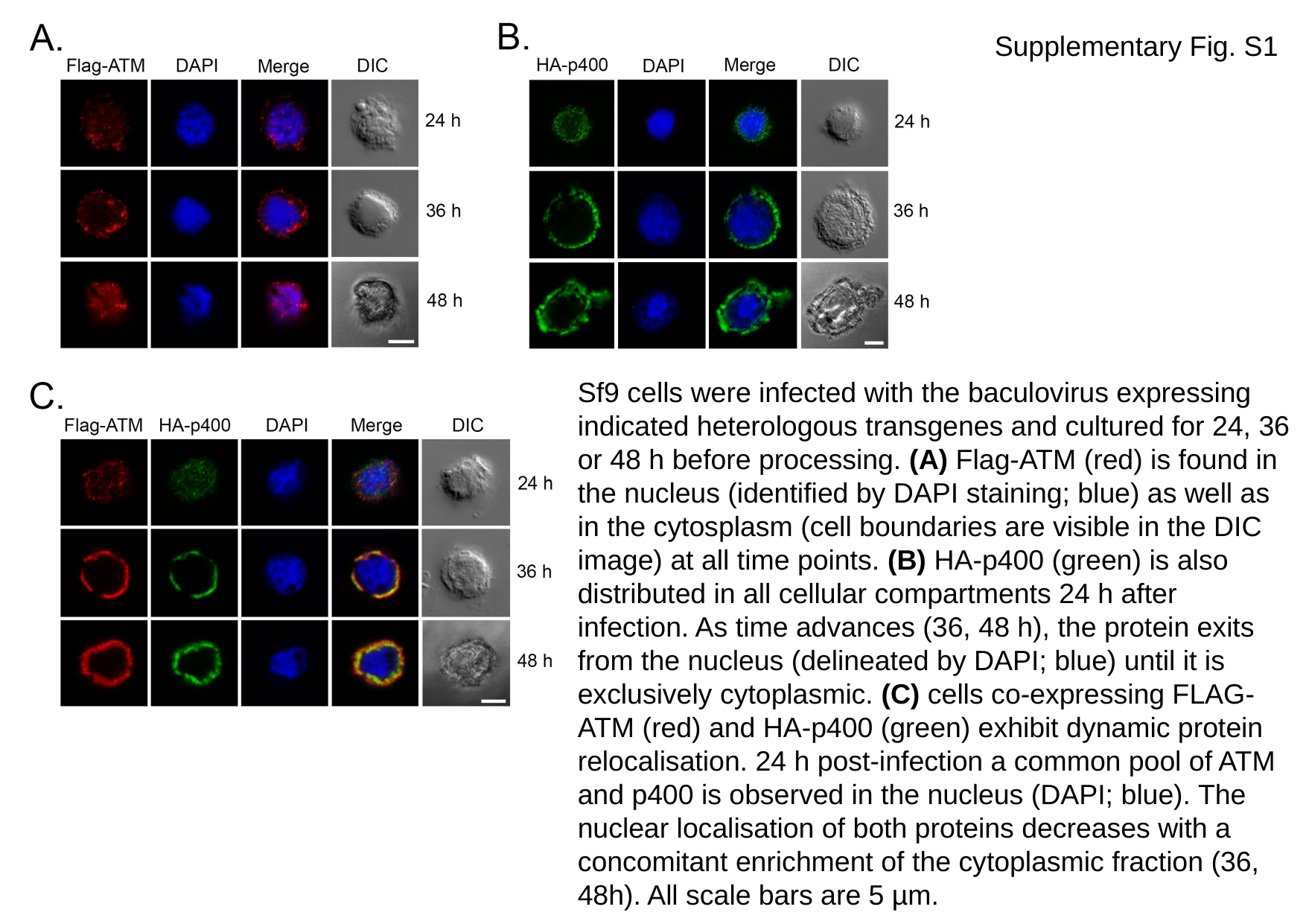

Supplementary Fig. S1
Sf9 cells were infected with the baculovirus expressing indicated heterologous transgenes and cultured for 24, 36 or 48 h before processing. (A) Flag-ATM (red) is found in the nucleus (identified by DAPI staining; blue) as well as in the cytosplasm (cell boundaries are visible in the DIC image) at all time points. (B) HA-p400 (green) is also distributed in all cellular compartments 24 h after infection. As time advances (36, 48 h), the protein exits from the nucleus (delineated by DAPI; blue) until it is exclusively cytoplasmic. (C) cells co-expressing FLAG-ATM (red) and HA-p400 (green) exhibit dynamic protein relocalisation. 24 h post-infection a common pool of ATM and p400 is observed in the nucleus (DAPI; blue). The nuclear localisation of both proteins decreases with a concomitant enrichment of the cytoplasmic fraction (36, 48h). All scale bars are 5 µm.
