# Supplementary material for: Ataxia telangiectasia mutated (ATM) interacts with p400 ATPase for an efficient DNA damage response
Source: BMC Mol Biol. 2016 Nov 4;17:22. doi: 10.1186/s12867-016-0075-7 (PMC5097431; doi:10.1186/s12867-016-0075-7)
Supplement: Supplementary file 2 — Additional file 2: Figure S2. (A) ectopic expression of ATM-interacting p400 fragments in U2OS cells. U2OS cells were infected with lentivirus expressing Flag-tagged p400 fragments and selected for 5 days against puromycin. Total 500 μg of protein was immunoprecipitated with M2 agarose and analysed by immunoblotting with anti-Flag antibody. (B) growth curve of puromycin-selected U2OS cells. U2OS cells were infected with lentivirus expressing Flag-F1or Flag-F6, and selected for 5 days against puromycin. Each cell line was seeded in 96-well plate in triplicate and monitored for the cell proliferation. [file 12867_2016_75_MOESM2_ESM.pptx]

## Slide 1
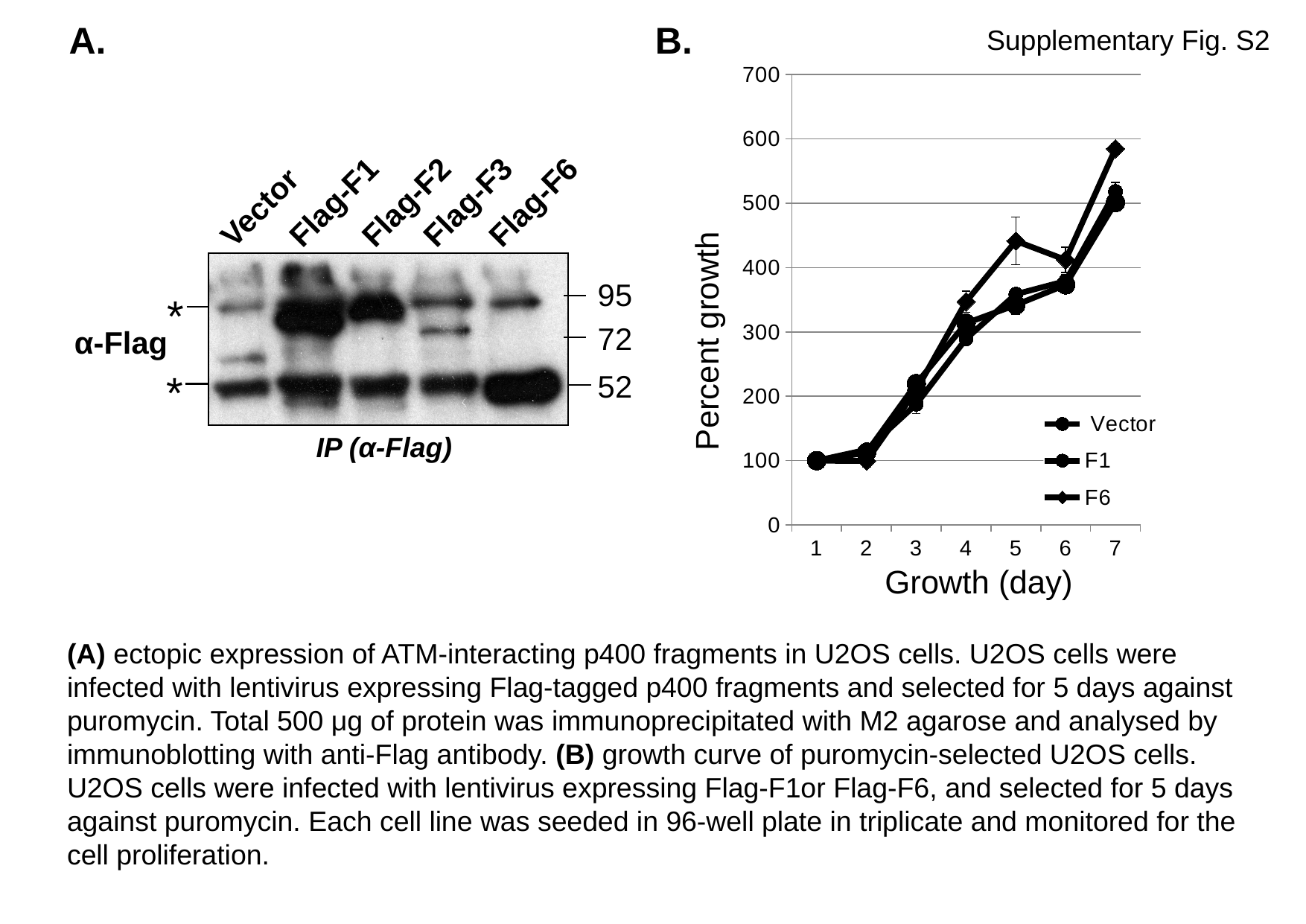

A.
B.
Supplementary Fig. S2
### Chart
| Category | | | |
|---|---|---|---|Flag-F1
Flag-F2
Flag-F3
Flag-F6
Vector
95
*
72
Percent growth
α-Flag
*
52
IP (α-Flag)
Growth (day)
(A) ectopic expression of ATM-interacting p400 fragments in U2OS cells. U2OS cells were infected with lentivirus expressing Flag-tagged p400 fragments and selected for 5 days against puromycin. Total 500 μg of protein was immunoprecipitated with M2 agarose and analysed by immunoblotting with anti-Flag antibody. (B) growth curve of puromycin-selected U2OS cells. U2OS cells were infected with lentivirus expressing Flag-F1or Flag-F6, and selected for 5 days against puromycin. Each cell line was seeded in 96-well plate in triplicate and monitored for the cell proliferation.
